# Thermophysical properties of a Si_50_Ge_50_ melt measured on board the International Space Station

**DOI:** 10.1038/s41526-020-0100-5

**Published:** 2020-03-25

**Authors:** Yuansu Luo, Bernd Damaschke, Georg Lohöfer, Konrad Samwer

**Affiliations:** 1 I. Physikalisches Institut, Georg-August-Universität, D-37077 Göttingen, Germany; 2 Institut für Materialphysik im Weltraum, Deutsches Zentrum für Luft- und Raumfahrt (DLR), D-51170 Köln, Germany

**Keywords:** Materials science, Structure of solids and liquids

## Abstract

Thermophysical properties of highly doped Si_50_Ge_50_ melt were measured contactlessly in the electromagnetic levitation facility ISS-EML on board the International Space Station. The sample could be melted, overheated by about 375 K, and cooled down in 350 mbar Argon atmosphere. A large undercooling of about 240 K was observed and a quasi-homogeneous nucleation on the droplet surface occurred. During the cooling phase, high-resolution videos were taken from the side and the top. The density and thermal expansion were evaluated with digital image processing; the viscosity and the surface tension were measured by means of the oscillating drop technique. Inductive measurements of the electrical resistivity were conducted by a dedicated electronics. All data were taken as a function of temperature *T* from the overheated melt down to the undercooled range. We found a nonlinear thermal expansion, suggesting a many body effect in the liquid beyond the regular pair interaction, an enhanced damping of surface oscillations likely related to an internal turbulent flow, and an increment of the electrical resistivity with decreased *T* in the undercooled range regarding a demixing of the components.

## Introduction

The semiconductors Si and Ge have been the workhorses for electronic hardware production for a long period of time. In addition, Si–Ge alloys and heterostructures using these alloys have come in the focus of interest due to their flexible electronic properties^1–4^. For productions and applications of materials, the knowledge of thermophysical properties of the melt is essential and they have to be measured accurately. For this purpose we started the ESA-project “SEMITHERM” (“Investigations of thermophysical properties of liquid semiconductors in the melt and the undercooled state under microgravity conditions”, ESA-AO-2000-068). The goals of the project include the precision measurements of the density, thermal expansion, surface tension, viscosity, and electrical resistivity of the liquid Ge and alloys Si_1−*x*_Ge_*x*_ (*x* = 0.75, 0.5, and 0.25). Due to the strong reactivity of the high-temperature melts the experiments need to be performed contactlessly under low-gravity conditions. The Electromagnetic Levitator (ISS-EML) on board of the International Space Station (ISS) is the instrument of choice. For the preparation of these experiments, measurements on parabolic flights have been performed in a similar low-gravity electromagnetic levitation facility (TEMPUS)^5^. The results for Ge-rich Si–Ge alloys have been reported in a previous paper^6^.

Here we report about the measurements with an Si_50_Ge_50_ sample on board the ISS. Reports about other concentrations will follow. First, we give an overview of the general processing of the sample, followed by the presentation of the results of measurements of the viscosity, surface tension, density, thermal expansion, and electrical conductivity. All properties were measured in the melt as a function of temperature. In the last section an overview of the EML facility, the experimental details including the sample properties is given.

## Results and discussion

### Melting, cooling, undercooling, and solidification behavior

Eleven melting and cooling cycles of the Si_50_Ge_50_ sample (always the same sample) were successfully carried out on board the ISS. Table 1 gives an overview of the processing designed for each cycle, including values of overheat, achieved undercooling and cooling rates (in 350 mbar Ar) above and below the liquidus temperature *T*_l_ (1548 K). The overheat is about 375 K in average. Undercooling of about 45 ± 10 K was obtained from cycles #1–#5, jumping to 155 K in cycle #6 and then gradually rising up to 240 K for cycle #11. The result observed arises possibly from gradual melting of the surface oxide SiO_2_ at *T*_max_, which is close to the melting point (1986 K) of SiO_2_^7^. In addition, silicon monoxide (SiO) may gradually form at high *T* via a decomposition reaction, SiO_2_ + Si ⇌ 2 SiO; SiO is volatile and can evaporate away by sublimation^8^.

**Table 1:** Overview of melting cycles performed for the sample Si_50_Ge_50_, including achieved overheat and undercooling as well as averaged cooling rates measured undercooling gas (350 mbar Ar).

Cycle	SCE	Pulses	Axial/radial camera (Hz)	Overheat (K)	Undercooling (K)	Averaged cooling rates (*T* > *T*_l_/*T* < *T*_l_) (K s^−1^)
#1	–	–	50/400	378	35	36/19
#2	–	–	50/400	365	40	35/20
#3	On	–	50/400	385	50	34/16
#4	On	–	50/400	355	35	31/12
#5	–	4×	150/400	357	55	30/13
#6	–	4×	150/400	366	155	29/10
#7	–	4×	150/400	380	200	30/7.5
#8	–	4×	150/400	375	190	30/7.8
#9	–	2×	150/400	390	200	28/7.8
#10	–	2×	150/400	390	205	29/7.5
#11	On	–	50/400	380	240	28/6

SCE is a sample coupling electronics used to determine inductively the sample resistivity (see Fig. 7).

**Fig. 1:**
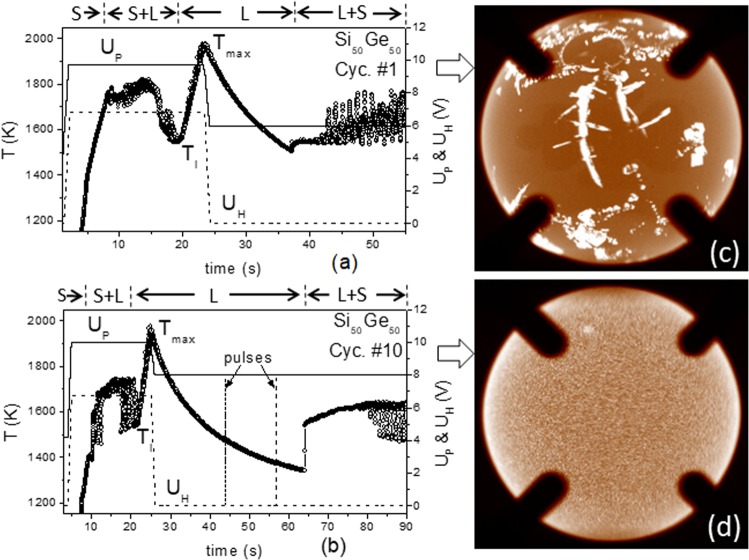
Time–temperature profiles (circles) measured for Si_50_Ge_50_. **a** from cycle #1 and **b** from cycle #10, showing a small and a large undercooling, respectively. Solid and dashed lines show the voltages *U*_P_ and *U*_H_ controlling the electromagnetic fields for positioning and heating of the sample. **b** shows two pulses used for excitation of drop oscillations. **c** and **d** are images of the sample recorded at beginning of the solidification, showing strongly heterogeneous and more homogeneous nucleation at the surface associated with small and large undercooling shown in (**a**) and (**b**).

As an example, Fig. 1a, b reveal time–temperature profiles *T(t)* measured by the radiation pyrometer for the cycles #1 and #10. The sample temperature *T* given here was calibrated using Wien’s law: *1/T* = *1/T*_p_ + *(1/T*_l_ −*1/T*_lp_*)*, where *T*_p_ is the pyrometer signal and *T*_l,p_ is the pyrometer signal at the liquidus temperature. In the beginning of the cycle the temperature reading of the solid sample rises quickly and reaches a plateau corresponding to the solidus temperature *T*_s_ (1383 K), where the sample starts to melt. The *T(t)-*profile approaching *T*_s_ shows no significant disturbances, indicating that in spite of a low electric conductivity the highly doped semiconductor can be well positioned in the EML facility. Because of the lower emissivity of the liquid, the temperature reading *T(t)* drops down during melting and subsequently reaches a minimum, which corresponds to the actual liquidus temperature *T*_l_. The large fluctuation (Δ*T* ≈ 200 K) of the *T(t)-*profile in the two phase region arises from the different spectral emissivity of the solid (0.48) and the liquid (0.22) read by the pyrometer depending on the actual spot. The small fluctuation (Δ*T* ≈ 50 K) is due to changes in view factor caused by movement of the sample. During melting the sample becomes metallic, so that the force applied by the electromagnetic levitation field on the droplet grows and improves its positioning stability. As a consequence, the *T(t)-*profile in liquid state appears very smooth. Figure 1c, d is the video image of the sample obtained at the beginning of solidification from cycle #1 and #10. These show a strongly heterogeneous and a more homogeneous nucleation at the surface corresponding to small and large undercooling degrees indicated in Fig. 1a, b, respectively. The strongly heterogeneous nucleation is accompanied by a dendritic growth (Fig. 1c) and emerges mainly in cycles #1–#5, where residual SiO_2_ oxide impurities on the surface serve likely as a nucleus for solidification. Because of a low height of free energy barrier, the heterogeneous nucleation is in general more common than homogeneous nucleation.

The rather homogeneous nucleation occurs for the first time in cycle #6, where the undercooling degree jumps up with no observable dendrite structure (Fig. 1d). The phenomenon can be contributed to the absence of the oxide impurities, which are obviously molten or evaporated during the processing in the first cycles, followed by a rise in undercooling (Table 1) in the subsequent cycles. Because of the homogeneous coverage of nuclei on the sample surface, the *T(t)-*profile now appears smooth at the beginning of the solidification and rises due to the exothermic nature of the process. The growth of the nuclei is accompanied by a volume expansion (volume anomaly^6^) and as a result, the liquid inside is partly pressured out, forming a bump on the surface. For this case, the *T(t)-*profile fluctuates regularly up and down upon the sample rotation.

### Oscillating drop method

**Fig. 2:**
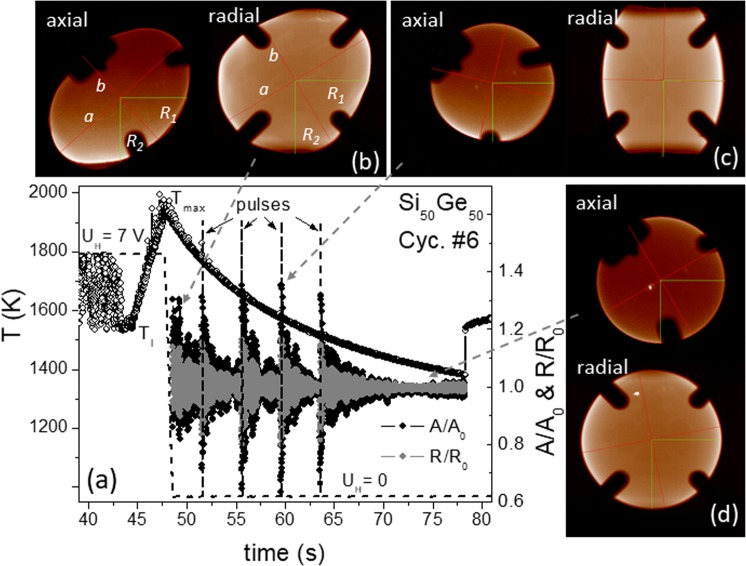
Time profiles and sample images of cycle #6. **a** Gives temperature (circles), voltage *U*_H_ (dashed line) controlling the electromagnetic heating field with four short pulses used for exciting droplet oscillations, as well as normalized areas *A/A*_0_ (black) and radii *R/R*_0_ (gray). Relevant axial and radial images of the sample captured between heating-off and the first pulse (**b**), at the third pulse (**c**), and far away from the fourth pulse (**d**), respectively, involving elliptical semiaxes *a*, *b* (red), horizontal and vertical radii *R*_1_, *R*_2_ (green) as well as areas *A* surrounded by edge curves.

Figure 2 shows typical axial and radial images measured for Si_50_Ge_50_ at different points indicated in the relevant *T(t)-*profile in Fig. 2a. The artefacts stem from parts of the sample holder. The areas were excluded in the edge fitting procedure, yielding data of areas *A* and lengths of horizontal and vertical radii *R*_1_ and *R*_2_ as well as elliptical semiaxes *a* and *b*, used for evaluation of thermophysical properties via the oscillating drop method. To minimize errors, we introduce an average radius *R* calculated from *R*_1_*, R*_2_*, a, b* and *r*_eff_, where *r*_eff_ is an effective radius extracted from the area *A* = *π r*_eff_^2^. The normalized areas *A/A*_0_ and radii *R/R*_0_ with *A*_0_ and *R*_0_ denoted as their mean values are plotted in Fig. 2a, showing damped oscillations caused by switching off the electromagnetic heating field, which also squeezes the droplet, and short voltage pulses.

**Fig. 3:**
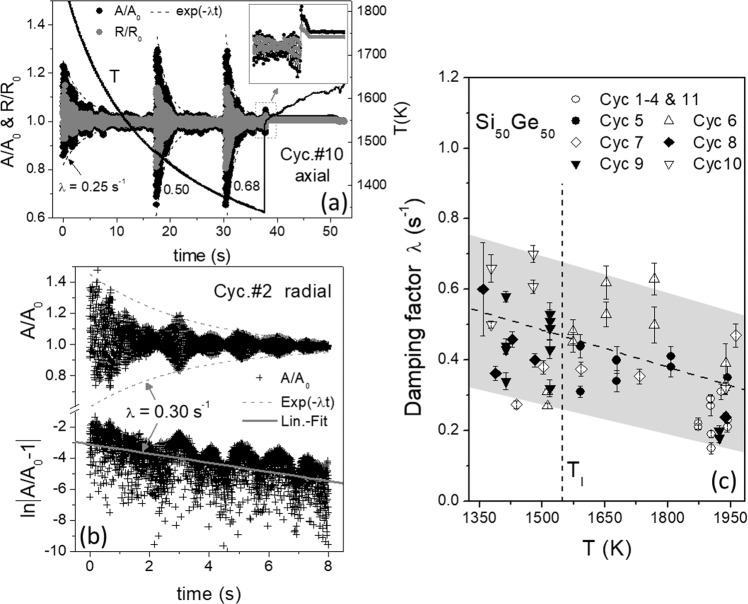
**a** *T(t)* profile of the cycle #10 with normalized axial areas *A/A*_0_ (black) and radii *R/R*_0_ (gray), revealing oscillations excited by heating-off und short pulses respectively and decayed with *t* in an exponential function e^−*λt*^ (dashed lines), as well as abrupt changes occurring upon solidification (inset) regarding volume anomaly^6^. **b** (upper) Decayed oscillations of radial *A/A*_0_ from cycle #2 excited by heating-off, showing an extra low period of ~1 Hz caused by droplet rotations, (bottom) linear fitting of ln|*A*/*A*_0_−1| to get the decay constant *λ*. **c** Damping factors *λ* of the oscillations measured for 11 cycles. A linear fit gives *λ* = 0.5–3.5 × 10^−4^(*T−T*_*l*_) s^−1^ (dashed line). The gray background indicates the scattering of ±0.2 s^−1^. The week *T* dependence is probably due to the influence of the turbulent flow effect.

As illustrated in Fig. 2c, the short pulse squeezes the drop into a prolate shape along the perpendicular axis. The cross-section viewed from the top in axial direction becomes correspondingly small and in radial direction looked incomplete due to a hiding effect of the magnetic coil (its winding distance is 8 mm). The uniaxially deformed droplet relaxes to its equilibrium spherical shape, giving rise to surface oscillations. A similar behavior appears at *T*_max_ by turning off the heating. In this case the uniaxial deformation is however much stronger than that induced by short pulses, giving rise to rotation of the droplet around an arbitrary axis with a period of about 1 Hz (see Fig. 3b). The overlap between rotation and oscillation makes the droplet temporarily aspherical (Fig. 2b) and lead to extra scattering of the experimental data (Fig. 5a). Only far away from heating-off or pulses, the droplet becomes quiet and well spherical, as shown in Fig. 2d. For this case, all length data *R*_1_*, R*_2_, *a,* and *b* are nearly identical and the scattering becomes smaller (Fig. 5b).

### Viscosity of the droplet

Typical oscillations of the levitated Si_50_Ge_50_ droplet excited at different *T* by start off and on heating pulses are presented as a function of the time *t* in Figs. 2a and 3a, b. The amplitude decays exponentially with *t* and approaches then the mean value *A*_0_ (or *R*_0_). The behavior can be fitted by *f* = *f*_0_ ± *Δf*·e^*−λt*^, here *f/f*_0_ is denoted as *A/A*_0_ or *R/R*_0_, Δ*f* as their maximal amplitude. The fit provides the envelope of the oscillation as plotted in Fig. 3a, b (dashed line) and yields a matched decay factor *λ*, which can be referred as the magnitude of the viscosity, i.e. *η* *=* *κλ*^9^. Here the constant *κ* = $\frac{{3M}}{{20r_0}}$ = 12.8 mPa s^2^ is calculated with *M* = 1.07 g and *r*_0_ = 4 mm.

The dashed lines in Fig. 3a illustrate the envelopes matched to oscillations (axial data of cycle #10) excited by heating off at 1940 K (*T*_max_) and two short pulses at 1480 and 1380 K. The relevant decay factors *λ* are found to be 0.25, 0.5, and 0.68 s^−1^, respectively, showing a temperature dependence. Figure 3b shows the oscillation of the droplet caused by heating off only (radial data of cycle #2). The impact from the rotation is visible. It creates a parasitic oscillation of about 1 Hz and disturbs the observation of the decay behavior, giving rise to additional errors. The dashed lines in Fig. 3b (upper part) present the fits with exponential function of e^−^^*λt*^, while the solid line in Fig. 3b (bottom) is simply obtained by linear fitting of the natural logarithm ln $\left| {A/A_0 - 1} \right|$. Both calculations yield the same decay constant λ. In this case 0.3 s^−1^ by fitting the data up to *t* = 8 s.

Figure 3c gathers the damping factors λ of the oscillating droplet Si_50_Ge_50_ at different *T* from data of cycles #1 to #11. They are lower than the values measured in parabola flights^6^ and only weakly temperature dependent. Note the oscillation of cycle#1–#4 and #11 is excited by heating-off at *T*_max_ only. The relevant decay constant *λ* was found to be within 0.15–0.31 s^−1^ (circles in Fig. 3c) and averaged at 0.23, which would correspond to a saturation value *η* of about 3.0 mPa s at high *T*. *λ* rises then slightly with decreasing *T*, passing through *T*_l_ without a remarkable change. At low *T*, it amounts to about 0.6 s^−1^, corresponding to *η* of about 8 mPa s.

For a discussion of the viscosity data measured here by the oscillating drop method the fluid flow in the sample plays a crucial role. In EML experiments even the positioning field in the cooling phase can lead to turbulent flow and therefore to an enhanced viscosity^10^. Qualitatively, the turbulent behavior can be observed by the movements of oxide particles, which exist on the surface in the first five cycles. Particles of about 50–100 µm in size could be tracked in the radial video images. They circulate or move chaotically on the droplet surface with maximum observed velocities v of about 0.12 m s^−1^. The Reynolds number *R*_*e*_, which is defined as the ratio of inertial force $\frac{{\rho V^2}}{L}$ to viscous force $\frac{{\eta V}}{{L^2}}$ (*ρ* is the density of the droplet and *L* its characteristic dimension), i.e. $R_e = \frac{{\rho VL}}{\eta }$^ 11^, can be estimated to be *R*_*e*_ ≥ 5700, taking *ρ* = 5.9 g cm^−3^, *L* = 8 mm (diameter of the sample) and the viscosity *η* assumed to be ≤1 mPa s as measured in experiments with an electrostatic levitator (ESL), where no turbulence should occur^10,12,13^. The high *R*_*e*_ value of the Si_50_Ge_50_ droplet causes the flow instability and turbulent flow occurs.

An additional indication for the turbulent flow is the dynamical behavior of the surface of the melt showing self-excited oscillations during the whole cooling phase. For doped Ge, which is used in another EML-experiment, fluid flow modeling resulted in high Reynolds numbers of about 6500–70,000 indicating highly turbulent flow within the droplet in the whole temperature range^14^.

These findings lead to the conclusion that the measured damping factor *λ* may be dominated by the levitation force induced fluid flow and as a result, the relevant *η* may deviate from the molecular viscosity measured in other experiments on earth. The argumentation is supported by the smaller value of <1 mPa s measured for Si in an ESL by Zhou et al.^12^ and Li et al.^13^. In contrast to an EML, the levitation force does not drive convection in an ESL. For previous EML experiments on parabola flights with Ge-rich Si-Ge-samples^6^, we measured viscosity data of about 5–10 mPa s at high temperatures, higher than the actual values measured on ISS, which may be caused by very high positioning fields necessary in parabola flights and therefore enhanced fluid flow.

**Fig. 4:**
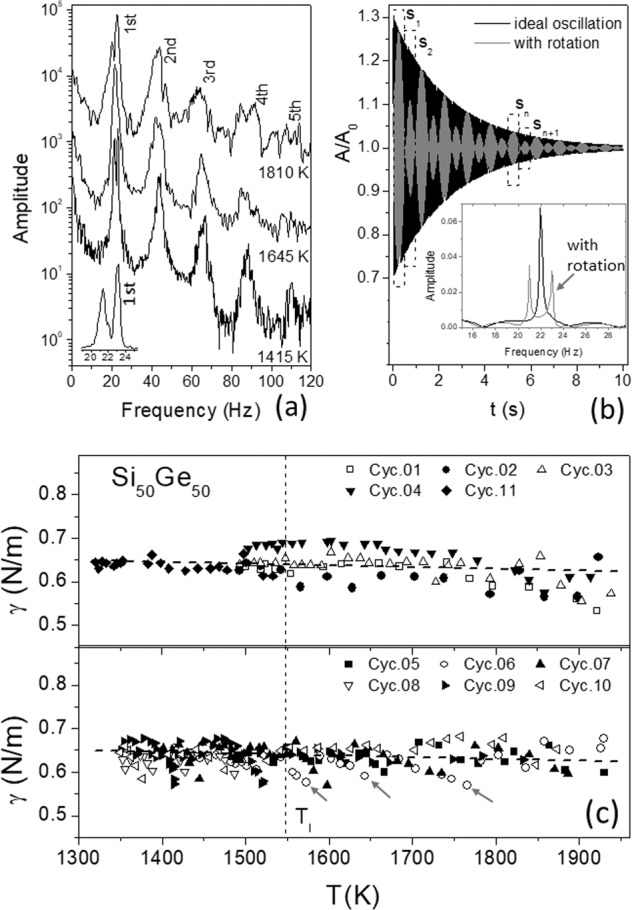
**a** Frequency spectra obtained from droplet oscillations induced by short pulses at 1810, 1645, and 1415 K, respectively. **b** Simulation of oscillations without and with droplet rotation. **c** Surface tension *γ* obtained (upper) from the cycles #1–#4 and #11, (bottom) from pulse cycles #5–#10, the lower values are indicated by arrows (here only for cycle #6) associated with relatively high droplet deformations at the beginning of excited oscillations.

In addition, the molecular viscosity *η* may be estimated roughly by its relation to the surface tension *γ* as suggested by Egry^15^ and Brillo^16^, namely $\frac{\gamma }{\eta } = 0.94\sqrt {\frac{{RT}}{W}}$. With the molar gas constant *R* = 8.314 J mol^−1^ K^−1^, the molecular weight *W* = 50.35 g mol^−1^ of Si_50_Ge_50_ and a measured value *γ* = 0.6 N m^−1^ near *T*_l_ (cf. Fig. 4a) we get a value *η* of about 1.2 mPa s in accordance with the measured values discussed. In principle, this form provides an opportunity to separate the temperature dependence of the damping into turbulent and laminar flow. On ther other hand it should be noted that the relation derived by Egry is not strictly applicable for Si_50_Ge_50_. It is only valid for pure elements and simple metallic liquids and should only be used for an estimate of the order of magnitude for the viscosity.

### Surface tension of the droplet

To determinate the surface tension *γ* of the droplet Si_50_Ge_50_, the frequency spectra were deduced by fast Fourier transform (FFT) of the oscillation data. Figure 4a exhibits the frequency spectra obtained from the droplet oscillations excited by short pulses at 1810, 1645, and 1415 K, respectively, exhibiting a resonance peak located at *ν* = 22 Hz and its higher harmonic peaks. A temperature dependence is here visible; the resonance peaks are shifted towards higher frequency for decreasing temperature. Since *γ* is a function of *ν*, namely *γ* =*ξv*^2^ (ref. ^9^) with *ξ* = $\frac{{3\pi M}}{8}$ (*M* is the droplet mass), this result implies an increase of *γ*. Parasitic oscillations arising essentially from the rotation of the droplet (~1 Hz) are overlapped with the surface oscillation, leading to splitting of the resonance peaks (detailed see the inset in Fig. 4a, b) and have to be taken into account by the evaluation of the resonance frequency.

To minimize the impact from the parasitic oscillations, we have to operate the FFTs segmentally using a series of time segmentations, s_1_,s_2_, … as schematically illustrated in Fig. 4b. By means of a careful division, a data segmentation yields a frequency spectrum with a single resonance peak (though it may appear somehow slightly asymmetrical). A series of *γ* values as a function of the temperature were extracted in this manner and are plotted versus *T* in Fig. 4c (where dashed lines are drawn in view of the average). The results in Fig. 4c (upper part) are from the cycles #1–#4 and #11 (without additional pulses), in which the droplet oscillations are induced by heating-off at *T*_max_ (cf. Fig. 3b) and driven by turbulent flow over whole temperatures until *T* < *T*_l_. Since the surface resonance selects natural frequency of the droplet^10^, the self-excited oscillation enables the measurements of the surface tension in the whole *T* range with similar results obtained from the pulse cycles #5–#10 (see Fig. 4c, lower part).

In addition to the errors caused by splitting or asymmetrical behavior of the resonance peak regarding the droplet rotation, the deformation of the droplet may give rise to a frequency shift, as recently investigated by Xiao et al.^17^, using a molten nickel-based superalloy with a deformation amplitude *δ* up to 7%. They gave an equation, $\frac{{\Delta v}}{v} = p_1\delta + p_2\delta ^2$, to describe the deformation induced frequency shift. Since the constant *p*_1_ and *p*_2_ are negative, the frequency shifts towards lower values. This nonlinear behavior is also visible here and even pronounced at the beginning of excited oscillations as indicated by arrows in Fig. 4c (lower part). The deformation amplitude *δ* of the molten Si_50_Ge_50_ can be estimated from the *R/R*_0_ data shown in Figs. 2 and 3a. It is about 15% at the beginning of the excited oscillations and decreases rapidly with *t*. After 1 or 2 s, *δ* < 10%. The *δ* value is somewhat above the limit reported in ref. ^17^. Nevertheless, the equation mentioned is valid for a qualitative discussion. An influence from oxide contamination (a few separated tiny particles) is insignificant, though it causes a small undercooling in the cycles #1–#5.

The measured surface tension is comparable to published data in the literature. In previous parabola flight experiments with undoped Si–Ge samples^18^ and doped Si–Ge samples with rich Ge content^6^, the values and the temperature dependences are similar. Measurements with an ESL also lie in the same range^13^. Experiments with the pendant drop method on earth show similar values^19^.

### Thermal expansion

**Fig. 5:**
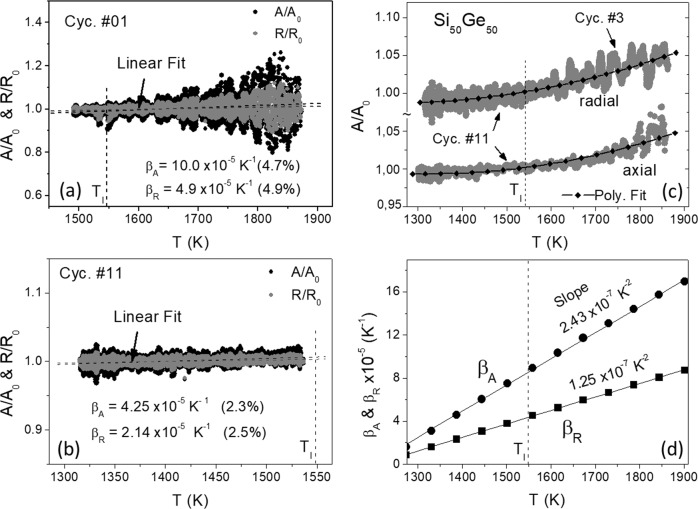
Normalized areas *A/A*_0_ and radii *R/R*_0_ measured as a function of *T* for Si_50_Ge_50_. **a**, **b** Radial data from cycle #1 and #11. **c** (bottom) Axial data from cycle #11 and (top) radial data from cycles #3 and #11, showing a nonlinear behavior, which can be fitted by a second-order polynomial. **d** Thermal expansion coefficients *β*_*A*_ (area) and *β*_*R*_ (radius) as a function of *T* with a slope in the order of magnitude of about 10^−7^ K^−2^.

Figure 5a shows the normalized *A/A*_0_ and *R/R*_0_ for the cycle #1. Largely scattered data beyond 1 ± 0.2 for *A/A*_0_ (1 ± 0.1 for *R/R*_0_) arising from strong deformations of the droplet during the heating-off period (about 1 or 2 s) were removed. Since *T* is here mostly above *T*_l_, the data represent behaviors of the overheated liquid. Figure 5b shows the data obtained from the deeply undercooled liquid (cycle #11 without a pulse). Due to a small deformation of droplet the scattering of the data is much smaller (1 ± 0.02), compared with those given in Fig. 5a.

Linear fitting these data yields coefficients *β*_*A*_ = *A*^−1^ d*A*/d*T* (*A* = area) and *β*_*R*_ = *R*^−1^d*R*/d*T* (*R* = radius) of area and linear thermal expansion in given temperature ranges. The error values in brackets shown in Fig. 5a, b give the uncertainty of the linear fit. As expected, *β*_*A*_ is found here to be nearly equal to 2*β*_*R*_. More interestingly, we found the coefficients (*β*_*A*_ = 10.0 × 10^−5^ K^−1^ and *β*_*R*_ = 4.9 × 10^−5^ K^−1^) obtained from the highly overheated melt (Fig. 5a), being much higher than those (4.25 × 10^−5^ K^−1^ and 2.14 × 10^−5^ K^−1^) obtained from the deeply undercooled one (Fig. 5b). The result suggests a nonlinear thermal expansion of the liquid.

To show this in more detail, Fig. 5c exhibits relevant axial and radial video data, covering both overheating and undercooling temperature ranges. Note that the radial data of cycle #11 is combined here with the radial data of cycle #3, due to the limited data storage capacities on board the ISS and thus a restricted length (≤30 s) of radial image video data (400 Hz). In addition, to minimize the scattering the data plotted in Fig. 5c were smoothed by adjacent averaging. A nonlinear behavior of the droplet size with *T* is apparent, which can approximately be fitted by a second-degree polynomial as is demonstrated in Fig. 5c. The polynomial has the form

1 $$R/R_{0} = \alpha_{R} + \beta_{R_{0}}\left( {T - T_{0}} \right) + \beta_{R} ^{\prime} \left( {T - T_{0}} \right)^{2}\,{\mathrm{and}}\,A/A_{0} = \alpha_{A} + \beta_{A_{0}}\left( {T - T_{0}} \right) + \beta _{A}^{\prime} \left( {T - T_{0}} \right)^{2}$$

with constant *α*_*R,A*_ close to 1. By differentiation of the Eq. (1), we get the thermal expansion coefficient as a linear function of *T*, namely

2 $$\beta _{R,A} = \beta _{R,A0} + 2\beta _{R,A}{^\prime}\left( {T - T_0} \right)$$

with *β*_*R,A*0_ ≈ 10^−5^ K^−1^ as initial value at *T*_0_ and *2β*_*R,A*_′ ≈ 10^−7^ K^−2^ as the slope (see Fig. 5d). With the help of the Eq. (2), the *β*_*R*_ value at the melting point *T*_l_ can be evaluated. Averaging over the cycles #1–#5, we get a mean *β*_*R*_ of 3.40 × 10^−5^ K^−1^. It appears larger than that (2.90 × 10^−5^ K^−1^) averaged over cycles #6–#11, which, in contrast to the cycles #1–#5, possess more data of the deeply undercooled and spherical droplet. The difference arises therefore, on the one hand, from fitting errors depending on the data precision, but on the other hand, it mirrors again the nonlinear behavior of the thermal expansion. The mean value *β*_*R*_ of 3.15 × 10^−5^ K^−1^ averaged using the values from all cycles represents the linear expansion coefficient of the Si_50_Ge_50_ melt near *T*_l_. It, although partly contributed from deep undercooled melt, appears slightly above the value of *β*_*R*_ = 3.0 × 10^−5^ K^−1^ measured by previous parabola flight experiments for overheated pure Ge (1275–1675 K)^6^. The result implies an impact of the composition. For the nonlinear term in the Eq. (1) we found 2*β*_*A*_′ = 2.43 × 10^−7^ K^−2^ and 2*β*_*R*_′ = 1.25 × 10^−7^ K^−2^ from the slopes in Fig. 5d. Again, a factor of ≈2 is found for *β*_*A*_′/*β*_*R*_′ (see above).

The density of the melts of Si and Ge was measured by a thermometric method by Glazov and Shcelikov^20^ about 20 years ago. They also found a nonlinear temperature dependence of the thermal expansion and discussed this behavior in terms of a decrease in the strength of interatomic bonds with increasing temperature. The experimental results are comparable to the behavior of the Si–Ge melts investigated here in the ISS-EML.

Kulkarni et al.^21^ performed ab initio MD simulations of Ge melts. They argue that at lower temperatures the structure factor shows a pronounced shoulder on the high *k*-side of the principal peak, which vanishes for high temperatures consistent with experimental scattering data in the literature. The coordination number increases with increasing temperature. This behavior of the structure factor is accompanied by an angle distribution function showing some tetrahedral arrangements at lower temperatures as well.

Following Kulkarni et al.^22^ our interpretation of the temperature dependence of the volume of the Si–Ge melt states that at very high temperatures the melt behaves like a simple metallic melt where a linear thermal expansion is expected^22^. With decreasing temperature, covalent, tetrahedral structure elements (small range order) begin to form and therefore the specific volume deviates from that of a simple liquid, which is characterized by a simple two body interatomic potential with linear thermal expansion, leading to the observed nonlinear temperature dependence.

In addition, the solidification of the melt is accompanied with an abrupt increase in *A/A*_0_ and *R/R*_0_ as illustrated in the inset of Fig. 3a. The phenomenon can be partly contributed to the volume expansion due to *T* rise, and partly to the volume anomaly of the substances Si and Ge, whose density in solid is lower than that in liquid^6^.

### Electrical resistivity of the droplet

The temperature-dependent electrical resistivity *ρ(T)* is a sensitive indicator for structural changes in the melt. Generally, the electrical resistivity in liquid metals results from the scattering of the conduction electrons at the disordered metal ions. Accordingly, the decline of the temperature dependent density fluctuations in the melt with decreasing temperature results in the usually observed linear decrease of *ρ*^22^. In liquid alloys a formation of compact structures (clusters) with decreasing temperature, due to chemical short-range ordering processes of the components, may result in an increase of the scattering cross-section for the electrons and thus of the electrical resistivity^23^. Consequently, an onset of clustering phenomena in a liquid alloy may lead to a deviation of *ρ(T)* from the typical linear temperature dependence. The lower the temperature of the melt is the more pronounced this effect should show up.

**Fig. 6:**
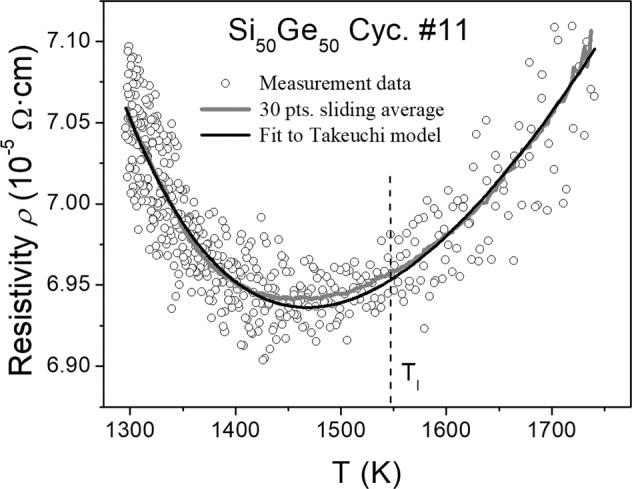
Inductively measured electrical resistivity data (circles) of the Si_50_Ge_50_ melt for different temperatures above the melting point and in the undercooled regime. The curve (black) is a fit of the resistivity model of Takeuchi and Endo^23^ to the data points, which agrees fairly well with the sliding average over 30 data points (gray line).

This above assumption is confirmed by the present liquid Si_50_Ge_50_ sample. Figure 6 shows a plot of the resistivity data against the temperature measured during the cooling phase of cycle #11, where the undercooling of the liquid Si_50_Ge_50_ droplet was very high (cf. Figure 1b and Table 1).

Assuming that the Si_50_Ge_50_ melt can be considered as a regular solution with a positive heat of mixing *W*^*Si,Ge*^ > 0^24^, meaning, that external heat is necessary to keep the liquid alloy components mixed, the unusual temperature behavior of the electrical resistivity can be described by the physical model of Takeuchi and Endo^23^

3 $$\rho ^{\mathrm {Si,Ge}}(x_{\mathrm {Si}},x_{\mathrm {Ge}},T) = \rho _0^{\mathrm {Si,Ge}}(x_{\mathrm {Si}},x_{\mathrm {Ge}}) + \rho _1^{\mathrm {Si,Ge}}(x_{\mathrm {Si}},x_{\mathrm {Ge}}) \cdot T + \frac{{\rho _2^{\mathrm {Si,Ge}}(x_{\mathrm {Si}},x_{\mathrm {Ge}})}}{{1 - 2\,x_{\mathrm {Si}}\,x_{\mathrm {Ge}}\,W^{\mathrm {Si,Ge}}/RT}}$$

with *x*_Si_ and *x*_Ge_ denoted as the concentrations of Si and Ge (presently *x*_Si_ = *x*_Ge_ = 1/2), $\rho _0^{\mathrm {Si,Ge}}(x_{\mathrm {Si}},x_{\mathrm {Ge}})$,$\rho _1^{\mathrm {Si,Ge}}(x_{\mathrm {Si}},x_{\mathrm {Ge}})$ and $\rho _2^{\mathrm {Si,Ge}}(x_{\mathrm {Si}},x_{\mathrm {Ge}})$ as temperature-independent constants. Corresponding to the conventional resistivity model, the Eq. (3) contains a constant term and a term which is linearly dependent on the temperature *T*. The last term finally considers the increase of the electrical resistivity with decreasing *T* due to the assumed increasing component demixing tendency in the melt, as discussed above. A weighted fit to the measured data points with *ρ*_0_^Si,Ge^ = 1.9 × 10^−5^ Ω cm, *ρ*_1_^Si,Ge^ = 2.2 × 10^−8^ Ω cm K^−1^, *ρ*_2_^Si,Ge^ = 1.6 × 10^−5^ Ω cm, and *W*^Si,Ge^ = 12 kJ mol^−1^ is shown in the diagram of Fig. 6. Even if the demixing assumption is too simple to explain the real structural development in the undercooled Si_50_Ge_50_ melt, Eq. (3) describes at least phenomenologically the measured temperature-dependent electrical resistivity of the Si_50_Ge_50_ melt fairly well. The structural evolution of the deeply undercooled Si_50_Ge_50_ melt may be also influenced by a liquid–liquid phase transition of the Si component and the reinforcement of its tetrahedral order^25,26^.

## Methods

### EML facility and sample description

**Fig. 7:**
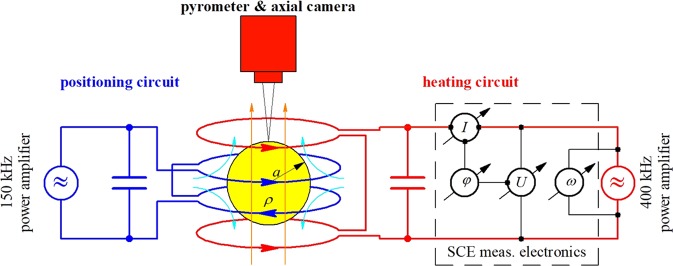
Principal sketch of the electrical oscillating circuits of the microgravity levitation facility ISS-EML including the quadrupole-like RF electromagnetic positioning field (cyan), and the homogeneous dipole-like RF electromagnetic heating field (orange). The corresponding electrical circuits are powered by their own current supply. The actual EML circuits, basing on the superposition principle, differ in details from those shown above. The SCE electronics, used to determine inductively the temperature-dependent sample resistivity and dimension, is hooked up to the heating circuit.

The experiments were performed in the microgravity electromagnetic levitator ISS-EML on board the International Space Station (ISS). The facility applies two superposed, radio frequency electromagnetic fields on electrically conducting samples in its center (see Fig. 7). A quadrupole-like “positioning field” (150 kHz), generated by two oppositely directed RF coil currents, to containerlessly confine a liquid droplet at a dedicated place against external residual forces, and a homogeneous dipole-like “heating field” (400 kHz), generated by two RF coil currents in the same direction, to efficiently heat and melt the sample inductively.

Although originally designed for metallic materials only, the low residual forces in the microgravity environment enable processing of highly doped semiconductors in the facility. A more detailed description of the levitation principle can be found in ref. ^27^. For visual observation of the samples two high-resolution CCD-cameras are installed. An axial camera with a frame rate of 50 Hz (resolution 704 × 704 pixels) or 150 Hz (resolution 352 × 352 pixels) looking from the top along the symmetry axis of the magnetic fields on the sample. This camera is combined with a pyrometer for temperature measurements. A radial camera with a frame rate of 400 Hz and a resolution of 600 × 600 pixels is looking from the side on the sample. More details of the facility can be found in ref. ^28^.

The spherical Si_50_Ge_50_ sample used has a mass of *M* = 1.07 g and a radius of *r*_0_ = 4 mm. The raw material stems from a highly doped alloy crystal prepared by the Czochralski technique in the group of Abrosimov^29^ at the IKZ Berlin. The dopant is boron (B) with a concentration of about 1.9 × 10^20^ atoms cm^−3^, which corresponds to an electrical conductivity of about 1.7 × 10^3^ Ω^−1^ cm^−1^ at 300 K. Due to the semiconducting behavior, the conductivity of the solid sample grows with *T* and jumps up to about 10^4^ Ω^−1^ cm^−1^ (corresponding to a resistivity of about 10^−4^ Ω cm as given in Fig. 6) upon melting regarding to the semiconductor–metal phase transition caused by disordering.

The sample concentration was checked/confirmed by EDX analyses. The liquidus temperature *T*_l_ at 1548 K and solidus temperature *T*_s_ at 1383 K are taken from the literature^30^.

### Ground test and parabola flight experiments

Relevant ground tests were made in advance by the Microgravity User Support Center (MUSC) at DLR, Cologne, Germany, to obtain the data of spectral emissivity *ε* required for the pyrometer, sample-coil coupling efficiency and the evaporation rate of the melt regarding the limits of dust generation. The measured spectral emissivity *ε* is 0.48 for the solid and 0.22 for the liquid phase. The inductive coupling efficiency is actually a measure for processability of the semiconductor sample in EML and was used for simulation of the planned melting cycles in ISS-EML. The coupling in the solid phase was found to be strong enough for a fast heating of the sample. In the liquid state, the sample is metallic and the coupling becomes enhanced by a factor of about 5.

The processability of highly doped semiconductors was verified by parabolic flight experiments provided by ESA and the DLR Institute of Material Physics in Space on board a Zero-G aircraft operated by Novespace, France using the similar EML setup TEMPUS^5^. The results indicated that the samples could be heated up in the solid phase, melted and positioned stable in liquid phase. Unfortunately, due to the short experiment time (~20 s) available and a relatively high *T*_l_ of the Si_50_Ge_50_ sample, only preliminary experiments could be performed.

### ISS experimental details

Before the transport to ISS, the sample was cleaned with acetone and ethanol in ultrasound bath and integrated in a special transport container which was pumped down to 10^−7^ mbar and filled then with 1 bar Ar. During the end inspection, the sample was exposed to air and it is expected that the surface is covered with a natural thin passivation oxide layer, which prevented the sample from further oxidation.

The ISS experiments were performed during the vibration-free astronaut sleep period with 11 cycles (Table 1), consisting of heating and melting to maximal temperatures of up to *T*_max_ ~1925 K and cooling down below *T*_s_, as shown in Figs. 1a, b and 2a. At *T*_max_ the heater was dimmed to its idle state, i.e. the heater control voltage was set to *U*_H_ = 0 and the positioner control voltage *U*_P_ decreased accordingly by about 2–3 V. During the following cooling phase, within which the disturbances by the electromagnetic force fields are just lowest, the different experiments with the sample were executed in an Argon gas atmosphere (350 mbar), which was necessary for a reduction of the evaporation rate (mass loss) of the overheated melt. A cycle took typically 1–2 min depending on the undercooling degree of the melt. A mass loss of about 0.1–0.2 mg per cycle was estimated from the evaporation experiments performed by MUSC as well as from the experience of the parabola flight experiments.

The surface tension γ and viscosity *η* of the melt were determined by the oscillating drop technique^9,28^. For this purpose, short pulses (triangle, 8 V, 0.05 s) in the heating field were set at different temperatures *T* in cycles #5–#10 (Figs. 1b, 2a, and 3a). The pulse squeezes the drop into a prolate shape along the heating field and results in damped surface oscillations to its equilibrium spherical shape. The exponential decay constant *λ* of the oscillation amplitude gives the viscosity of the sample, i.e. *η* = *κλ* with the proportional constant *κ =*$\frac{{3M}}{{20\pi r_0}}$ (*M* is the mass and *r*_0_ is the radius of the sample), while the resonance frequency *ν*, which can be extracted from the fast Fourier transform (FFT) of the oscillation data, yields the surface tension *γ* = *ξν*^*2*^ with *ξ* = $\frac{{3\pi M}}{8}$ (refs. ^9,31^). Unfortunately, the relaxation of the deformed drop is accompanied by a translational movement and a rotation (~1 Hz) of the droplet, which disturb the evaluation of the oscillation behavior and create extra scattering of the data. To minimize this disturbance, the melting cycles #1–#4 and #11 were done without pulses (cf. Table 1). However, at high temperatures near *T*_max_ the oscillation caused by switching off the heating field is unavoidable, leading to a large scatter of image data in this regime. The droplet oscillations were monitored by the axial and radial video cameras and its images were analyzed with respect to the above described oscillating drop method using a dedicated software “TeVi” (SEA Datentechnik GmbH), which detects the edges of the sample from the image contrast. The edge cure is fitted with a polynomial of degree 9 (ref. ^32^) and yields areas, radii, and semiaxes of the droplet. From these data also the thermal expansion coefficients *β* of the sample could be determined.

The electrical resistivity of the liquid Si_50_Ge_50_ droplet is measured inductively. For this purpose the homogeneous radio frequency (RF) magnetic heating field can very well be used. This field, generated by an RF current *I* of angular frequency *ω* in the heating coil (see Fig. 7) induces eddy currents in the material, the strength of which depends on the electrical resistivity of the droplet. These eddy currents in turn generate an RF magnetization field depending on the resistivity and dimension of the sample. It induces an additional voltage *U* backwards in the coil, the strength and phase shift *φ* (relative to the current *I*) of which is determined by a dedicated measurement electronics (SCE). The data are monitored with a rate of 10 Hz and with a resolution of 16 Bit, for details see ref. ^27^.

## Supplementary information

reporting-summary

## Data Availability

The datasets generated and analyzed during the current study are available from the corresponding author upon reasonable request.
